# Bisphosphonates enhance antitumor effect of EGFR-TKIs in patients with advanced EGFR mutant NSCLC and bone metastases

**DOI:** 10.1038/srep42979

**Published:** 2017-02-17

**Authors:** Guowei Zhang, Ruirui Cheng, Zengli Zhang, Tao Jiang, Shengxiang Ren, Zhiyong Ma, Sha Zhao, Caicun Zhou, Jun Zhang

**Affiliations:** 1 Department of Internal Medicine, The Affiliated Cancer Hospital of Zhengzhou University, Henan Cancer Hospital, Zhengzhou, 450003, China; 2 Department of Respiratory Medicine, The First Affiliated Hospital of Zhengzhou University, Zhengzhou, 450052, China; 3 Department of Medical Oncology, Shanghai Pulmonary Hospital & Thoracic Cancer Institute, Tongji University School of Medicine, Shanghai, 200433, China; 4 Department of Respiratory, The Second Affiliated Hospital of Soochow University, Suzhou, 215004, China; 5 Division of Hematology, Oncology and Blood & Marrow Transplantation, Department of Internal Medicine, Holden Comprehensive Cancer Center, University of Iowa Carver College of Medicine, Iowa City, Iowa

## Abstract

Whether bisphosphonates could enhance the effect of epidermal growth factor receptor (EGFR)-tyrosine kinase inhibitors (TKIs) in non-small-cell lung cancer (NSCLC) patients with EGFR mutation and bone metastases (BM) remains unknown. EGFR mutation status were collected from 1560 patients with NSCLC and BM. 356 NSCLC patients with EGFR mutation and BM were identified. Among them, 91 patients received EGFR-TKIs alone and 105 patients received EGFR-TKIs plus bisphosphonates as first-line therapy. Comparing to TKIs alone, EGFR-TKIs plus bisphosphonates had a statistically significant longer progression-free survival (PFS: 11.6 vs. 9.3 months; HR = 0.68, P = 0.009), while a similar overall survival (OS: 20.5 vs. 19.5 months; HR = 0.95, P = 0.743) in patients with EGFR-mutant NSCLC and BM. The incidence of skeletal-related events in combined group was numerically lower than that in EGFR-TKIs alone group (29.7% vs. 39.4%, P = 0.147). In multivariate analysis, EGFR mutation was found to be a significant independent prognostic factor for OS in NSCLC patients with BM (HR = 0.710, P = 0.021). In conclusion, EGFR mutation was the significant independent prognostic factor for OS and the addition of bisphosphonates to EGFR-TKIs could enhance the antitumor effect of EGFR-TKIs in patients with EGFR-mutant NSCLC and BM.

Lung cancer is one of the most common cancers as well as the leading cause of cancer-related death worldwide^1^,^2^. The averaged 5-year survival rate of lung cancer is 17.4%, which drastically reduced to 4.2% in distant or metastasized lung cancer^3^. Bone metastasis (BM) represents one of the most deleteriously metastatic lung cancer and associates with dismal prognosis. About 30–40% patients with non-small cell lung cancer (NSCLC) develop BM with a median survival of 6 months^4^,^5^,^6^. The skeletal-related events (SREs) caused by BM could further deteriorate the prognosis of patients with BM^5^,^7^,^8^.

Currently, the therapeutic strategies to manage BM are still limited. Systemic therapies that block osteoclast activity including bisphosphonates (zoledronic acid, ibandronic acid or disodium pamidronate etc.) and Receptor Activator of Nuclear factor Kappa B Ligand (RANKL) inhibitors (denosumab) can reduce the incidence of SREs^9^,^10^,^11^. In a phase III randomized trial, denosumab could slightly delay the appearance of SREs in NSCLC as compared to bisphosphonates^12^,^13^. Bisphosphonates are stable analogues of inorganic pyrophosphate that induce osteoclast apoptosis, inhibit bone resorption, osteoclast formation and recruitment. They are efficient for the prevention of skeletal complications and generally recommended in patients with BM. In the preclinical studies, bisphosphonates exerted anti-tumor effects in NSCLC, including the inhibition of tumor cell proliferation, invasion, angiogenesis, and micrometastasis^14^,^15^,^16^,^17^. Furthermore, preclinical data also showed that bisphosphonates could enhance the inhibitory effects of EGFR-TKIs on NSCLC with EGFR mutation both *in vitro* and *in vivo*^18^. In clinical setting, a retrospective study with a small cohort of 62 cases found that bisphosphonates plus EGFR-TKIs had statistically significantly longer progression-free survival (PFS) and OS than EGFR-TKIs alone (median PFS: 15.0 vs 7.3 months, P = 0.03; OS: 25.2 vs 10.4 months, P = 0.0015)^19^. However, this study could just present a clue of bisphosphonates on EGFR TKI due to the limited cases and larger cohorts study is needed to further validate the role of bisphosphonates in patients with EGFR mutant NSCLC and BM.

Thus, we analyzed this large cohort of 1560 patients with BM and advanced NSCLC to investigate whether bisphosphonates affect the outcome of first-line EGFR-TKIs therapy in Chinese NSCLC patients with EGFR mutation and BM. Meanwhile, we also assessed the role of bisphosphonates in advanced NSCLC patients who receiving chemotherapy as the first-line setting. In addition, the epidemiology and clinical characteristics of NSCLC patients with BM were analyzed.

## Results

### Patients characteristics

**Figure 1:**
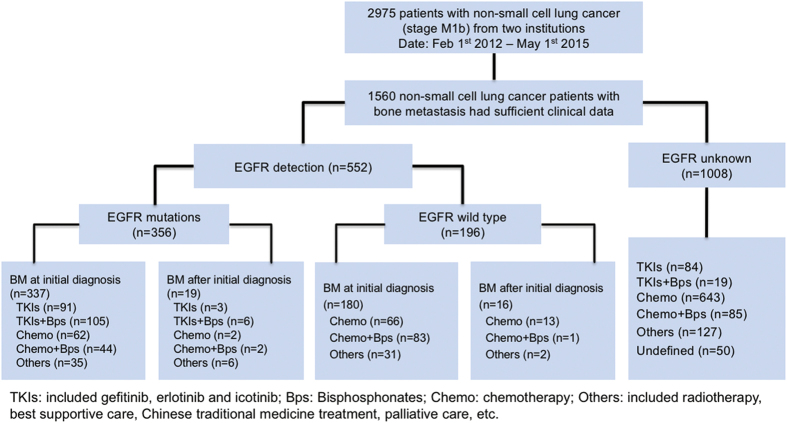
Flow chart of patient cohort.

A total of 2975 NSCLC patients with M1b stage were initially identified and 1560 NSCLC patients with BM enrolled into further analysis in this study from February 2012 to May 2015 (Fig. 1). Of them, 552 patients detected EGFR mutation status and 356 patients (64.5%) had EGFR mutations. 94 of the patients with EGFR mutations received EGFR-TKIs alone as first-line treatment and 111 of them received EGFR-TKIs plus bisphosphonates as first-line treatment. The clinical characteristics of the study population were summarized in Table 1. In brief, 264 (47.8%) were female and 288 (52.2%) were male patients were included, and the mean age was 60 years. Most of them were never smokers (71.7%), adenocarcinoma (88.2%) and had performance status of ECOG ≤ 1 (91.3%).

### Epidemiology of NSCLC patients with EGFR mutation and BM

552 patients underwent the EGFR detection (Fig. 1). There are 295 (53.4%) patients with EGFR common mutations including 143 (25.9%) patients with EGFR 19DEL and 152 (27.5%) patients with EGFR L858R. 61 patients had EGFR rare mutations. The major clinicopathological characteristics of the 552 patients with EGFR mutation tested are presented in Supplementary Table S1. The average age of patients with EGFR common and rare mutations were 59.9 and 58.1 years (P = 0.242). EGFR common mutations in NSCLC patients with BM were significantly associated with never-smoking, female gender and histology of adenocarcinoma (P = 0.000, P = 0.000, P = 0.000; respectively). EGFR rare mutations were significantly associated with female gender (P = 0.000) but not never-smoking and histology of adenocarcinoma (P = 0.061, P = 0.060; respectively). The major clinicopathological characteristics of all included BM patients are listed in Table 1.

### The efficacy of different therapeutic groups

**Figure 2:**
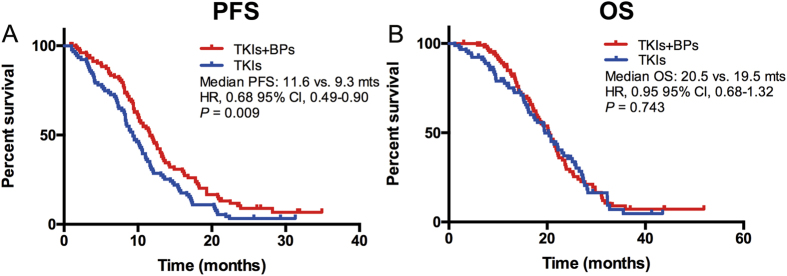
EGFR-TKIs plus bisphosphonates showed longer PFS (**A**) but similar OS (**B**) than those accepted EGFR-TKIs alone in NSCLC patients with EGFR mutation and bone metastasis.

**Figure 3:**
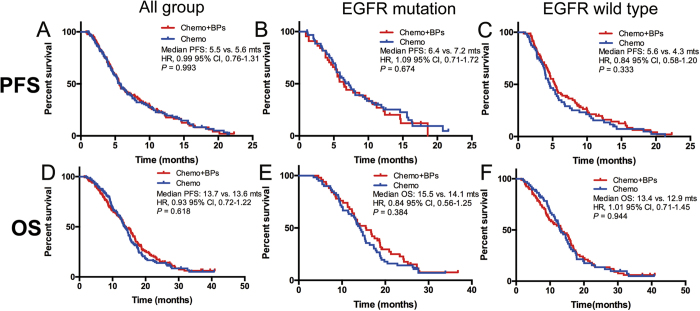
The effect of chemotherapy on survival in patients with NSCLC and BM. (**A,D**) Chemotherapy plus bisphosphonates showed similar PFS and OS than chemotherapy alone in patients with NSCLC; (**B,E**) Chemotherapy plus bisphosphonates showed no superior PFS and OS than chemotherapy alone in NSCLC patients with EGFR mutations and BM; (**C,F**) Chemotherapy plus bisphosphonates showed no superior PFS and OS than chemotherapy alone in NSCLC patients with EGFR wild type and BM.

In EGFR mutation group, 94 patients with BM received EGFR-TKIs and 111 patients received EGFR-TKIs plus bisphosphonates (Table 1). 91 patients with BM at the initial diagnosis received EGFR-TKIs and 105 cases received EGFR-TKIs plus bisphosphonates were included into the efficacy analyses. Tumor responses in different therapeutic groups were listed in Supplemental Table S2. Both the objective response rate (ORR) and disease control rate (DCR) were similar in EGFR-TKIs plus bisphosphonates vs. EGFR-TKIs alone groups (ORR: 58.1% vs. 48.9%, P = 0.290; DCR: 89.5% vs. 87.9%, P = 0.722). PFS was significantly longer in EGFR-TKIs plus bisphosphonates group than in EGFR-TKIs alone group (median PFS: 11.6 vs. 9.3 months; HR = 0.68, 95% CI 0.49–0.90, P = 0.009) (Fig. 2A). However, OS was similar in two groups (median OS: 20.5 vs. 19.5 months; HR = 0.95, 95% CI 0.68–1.32, P = 0.743) (Fig. 2B). The incidence of skeletal-related events in combined treatment group was numerically lower than that in EGFR-TKIs alone group (29.7% vs. 39.4%, P = 0.147). Chemotherapy plus bisphosphonates showed similar PFS (median PFS: 5.5 vs. 5.6 months; HR = 0.99, 95% CI 0.76–1.31, P = 0.993) and OS (median OS: 13.7 vs. 13.6 months; HR = 0.93, 95% CI 0.72–1.22, P = 0.618) than chemotherapy alone (Fig. 3A,D) in patients with NSCLC and BM. In the advanced NSCLC patients with EGFR mutation and BM who received chemotherapy as the first-line setting, the median PFS and OS were similar in both groups (median PFS: 6.4 vs. 7.2 months, HR = 1.09, 95% CI 0.71–1.72, P = 0.674; median OS: 15.5 vs 14.1 months, HR = 0.84, 95% CI 0.56–1.25, P = 0.384) (Fig. 3B,E). Among the patients with EGFR of wild type and BM at the initial diagnosis, 66 of them accepted chemotherapy and 83 patients accepted chemotherapy plus bisphosphonates. Chemotherapy plus bisphosphonates also showed similar PFS (median PFS: 5.6 vs. 4.3 months; HR = 0.84, 95% CI 0.58–1.20, P = 0.333) and OS (median OS: 13.4 vs. 12.9 months; HR = 1.01, 95% CI 0.71–1.45, P = 0.944) than chemotherapy alone (Fig. 3C,F).

### Univariate and multivariate analysis on overall survival

In univariate analysis of 552 patients with BM, patients without SREs had significantly better OS than those with SREs (HR = 0.764; 95% CI, 0.382–0.921; P = 0.044). Patients with ECOG PS 0–1 had significantly better OS than those presenting with ECOG PS > 1 (HR = 0.352; 95% CI, 0.179–0.811; P = 0.018) (Table 2). No significant difference was found in OS based on age (HR = 0.798, P = 0.263), smoking history (HR = 0.921, P = 0.255), histology of adenocarcinoma (HR = 0.898, P = 0.449), number of BM (HR = 0.889, P = 0.983) and extra-bone metastasis (HR = 0.545, P = 0.289) (Table 2). Of note, patients with EGFR mutation had significantly better OS than those with EGFR wild type (HR = 0.632; 95% CI, 0.497–0.805; P = 0.003). In multivariate analyses, ECOG PS remained the independent predictors of OS. BM patients with EGFR mutations had a significantly lower risk of death than those without (HR = 0.710; 95% CI, 0.597–0.913; P = 0.021) (Table 2).

## Discussion

To our best knowledge, the current study is the first large-scale observational study to investigate the epidemiology and role of the third-generation bisphosphonates in NSCLC patients with BM. 1560 NSCLC patients with BM were included into this study and 552 cases entered the epidemiological analysis of EGFR mutation and survival analysis. We found that EGFR mutation was a significant independent prognostic factor for OS in BM patients with NSCLC. More importantly, the addition of bisphosphonates to EGFR-TKIs had longer PFS, while similar OS than TKIs alone in BM patients with EGFR mutations. However, bisphosphonates showed no additional PFS or OS benefit when combined with chemotherapy as the first line setting in patients with NSCLC and BM.

Bisphosphonates are the frequently used for preventing SRE in NSCLC patients with BM^5^,^7^. Several preclinical studies found that bisphosphonates could enhance the effects of EGFR-TKIs on EGFR mutant NSCLC both *in vitro* and *in vivo*^18^,^20^. In clinical setting, a small cohort study with limited cases also showed that EGFR-TKIs plus bisphosphonates could significantly prolong PFS and OS in patients with EGFR mutant NSCLC and BM than those received EGFR-TKIs alone as the first-line therapy (PFS: 15.0 vs 7.3 months, P = 0.03; OS: 25.2 vs 10.4 months, P = 0.0015)^19^. In line with these studies, the present study also found that EGFR-TKIs plus bisphosphonates could prolong PFS than EGFR-TKIs alone (mPFS: 11.6 vs 9.2 months, P = 0.006). As we know, bisphosphonates can inhibit ERK1/2, AKT and signal transducer and activator of transcription 3 (STAT3) activation, which was also the direct downstream pathway of EGFR. Thus, bisphosphonates could have a synergetic mechanism with EGFR-TKIs^18^,^20^. The other possible explanation might be that the incidence rate of SREs in the TKI + BP group was numerically lower than it in the TKI group, which will contribute to a superior PFS in TKI + BP group since SREs are associated with poorer prognosis in patients with NSCLC and BM. However, OS was similar between two groups. In the past few years, dramatic improvement has been achieved for patients who get acquired resistance of EGFR-TKI due to the implementation of novel therapeutic strategies such as local therapies, anti-angiogenesis and third-generation EGFR-TKI. The patients enrolled into this study were chronological later than the patients in the previous study^19^. Thus, the treatment beyond the disease progression of EGFR-TKI might contribute the discrepancy.

Systemic chemotherapy is still the main therapeutic choice for advanced NSCLC without driver mutations. Several preclinical studies indicated that bisphosphonates could induce tumor cells apoptosis, inhibit tumor cell invasion and metastasis, affect immune microenvironment and therefore have antitumor effects in a variety of tumors, including NSCLC^18^,^19^,^21^,^22^. While in clinical setting, a recent randomized phase II study failed to show the ability of zoledronic acid to augment the cytotoxic effects of the combination of docetaxel/carboplatin and to delay disease progression in patients with inoperable stage IIIB or IV NSCLC^23^. Another phase II trial also demonstrated that the combination of bisphosphonates and docetaxel had a similar survival when compared with docetaxel alone in advanced NSCLC patients with BM^24^. Consistent with these results, our study demonstrated that chemotherapy plus bisphosphonates had a similar PFS and OS than chemotherapy alone, which suggest that bisphosphonates might not have a synthetic antitumor effect when combining with chemotherapy.

Comprehensive understanding the process of BM formation could help to develop potential novel therapeutic compounds for BM. Bone metastasis is a continuous and complicated process and several features of the bone marrow microenvironment involve, including interaction with osteoclasts and osteoblasts promoting bone degradation releasing extracellular matrix (ECM) bound growth factors, which could also promote the development of lung cancer micrometastases^25^,^26^. As a sequence, bone destruction and metastatic outgrowth, in another word “vicious cycle”, will continuously happen. One of the key factor to this vicious cycle is the RANKL^27^. Recently, a phase III trial demonstrated that denosumab (RANKL antibody) could prolong OS versus zoledronic acid in patients with NSCLC and BM^13^. RANKL’s major downstream target of is nuclear factor-kappa B, which is the downstream signaling pathway of EGFR^8^,^28^. Herein, it is worthwhile to explore whether EGFR-TKIs combined with RANKL target agents could also have a synergistic effect in patients with EGFR mutant NSCLC and BM. In addition, it was found that cellular and innate immunity in tumor microenvironment (TME) play pivotal roles in the modulation of BM^25^. For example, infiltration of myeloid derived suppressor cells (MDSC) could further stimulate tumor-induced osteolysis^29^. The role of other components in TME such as CD8 + T cells, regulatory T cells (Treg), programmed death 1 (PD-1), PD-L1 and etc. need to be further investigated to help guiding the application of immunotherapy in patients with NSCLC and BM.

Our study has several limitations that should be acknowledged. Firstly, despite the fact that the initial population was large, the number of patients who entered the final analysis was relatively small. Therefore, the findings in this study need to be further validated in prospective trials with large scale. Secondly, this study is a retrospective study, which will inevitably have selection bias. Due to the side effects of bisphosphonates, it was not recommended to some patients with renal dysfunction or dental diseases. Thirdly, all of patients in this study received the third-generation bisphosphonates (zoledronic acid and ibandronic acid). Therefore, whether other bisphosphonates could improve patient’s survival combined with EGFR-TKIs needs further investigation.

In conclusion, this study demonstrated that EGFR mutation was a significant independent prognostic factor for OS in patients with advanced NSCLC and BM. The addition of bisphosphonates to EGFR-TKIs could enhance the antitumor effect of EGFR-TKIs in patients with EGFR mutant NSCLC and BM, which suggested that third generation of bisphosphonates should be recommended in patients with advanced EGFR mutant NSCLC and BM who received EGFR-TKIs as the first-line setting.

### Patients and methods

#### Patients’ cohort

Patients with pathologically confirmed lung cancer at the Department of Medical Oncology, Shanghai Pulmonary Hospital and Henan Cancer Hospital from February 2012 to May 2015 were retrospectively collected. The lung cancer diagnosis was confirmed pathologically according to World Health Organization (WHO) pathology classification. Firstly, NSCLC patients with BM were selected. Then, patients received EGFR mutation test were chosen for the subsequent analysis. The major clinicopathological characteristics including demographic information, Eastern Cooperative Oncology Group performance status (ECOG PS), smoking history, lung cancer histology (WHO classification^30^, number of BM, sites of extra-bone metastasis, EGFR mutation status, time of BM diagnosis, and SREs were all collected. A never smoker was defined as a person who had smoked less than 100 cigarettes during his lifetime. Smoking status, ECOG PS and age were documented at the time of diagnosis. Thoracic Cancer Institute, Tongji University School of Medicine established requirements for clinical information on patient follow-up under treatment, including response to treatment and survival. EGFR common mutations were defined as mutations including exon 19 deletion (19DEL) and Leu858Arg point mutation in exon 21 (L858R). EGFR rare mutations were those mutations other than 19DEL and L858R. This study was approved by the ethics committee of Shanghai Pulmonary Hospital and Henan Cancer Hospital. We confirm that all methods were performed in accordance with the relevant guidelines and regulations. The written informed consent was obtained from each participant to use the clinical data for research before the medical intervention started.

### Molecular analysis

All mutational analyses were performed at the Thoracic Cancer Institute, Tongji University Medical School, Shanghai. Briefly, DNA from tissue was extracted using the DNeasy Blood and Tissue Kit or the QIAamp DNA FFPE Tissue Kit (both from Qiagen, Hilden, Germany). EGFR mutations were tested by amplification refractory mutation system (ARMS) as described in our previous studies (Amoy Diagnostics Co. Ltd., Xiamen, China)^31^,^32^,^33^,^34^,^35^.

### Treatment and assessments

The EGFR-TKIs used in this study included gefitinib (250 mg, once a day), erlotinib (150 mg, once a day) and icotinib (125 mg, three times a day). Pemetrexed (500 mg/m^2^), vinorebine (25 mg/m^2^; days 1 and 8) or gemcitabine (1000 mg/m^2^; days 1 and 8) was administered in combination with platinum-based chemotherapy every 3 weeks for 4–6 cycles until progressive disease (PD) or unacceptable toxic effect. Patients achieving complete response (CR), partial response (PR), stable disease (SD) would receive maintenance therapy. The decision whether to start bisphosphonates in NSCLC patients with BM were at the discretion of the treating physician. Zoledronic acid (4 mg) and ibandronic acid (6 mg) were administrated every 3–4 weeks according to the guidelines up to 2 years or until unacceptable toxic effect^36^,^37^. Bisphosphonates were concomitantly used with EGFR-TKIs or intravenously the other day of chemotherapy administration to avoid the simultaneously renal impairment. Tumor response was evaluated according to the Response Evaluation Criteria in Solid Tumors (RECIST) version 1.1, including CR, PR, SD or PD. The treatment response was evaluated one month after the initiation of therapy and then every 2 months.

### Statistical analysis

The categorical variables were compared using chi-square tests, or Fisher’s exact tests when needed. The continuous variable was analyzed by ANOVA and Tukey’s multiple comparison tests. Kaplan-Meier curve and two-sided log-rank test were used for univariate survival analyses. Cox proportional hazards model was used for uni- and multivariate survival analyses to calculate the hazard ratios (HR) and corresponding 95% confidence intervals (CI). OS was calculated from the date of lung cancer diagnosis to death from any cause or was censored at the last follow-up date. PFS was defined as the time from the date of first-line treatment initiation to the date of systemic progression or death and was censored at the date of last tumor assessment (when carried out). Disease progression was defined in accordance with the RECIST version 1.1. P values were two-sided and considered significant if less than 0.05. All statistical analyses were performed using the SPSS statistical software, version 20.0 (SPSS Inc., Chicago, IL, USA).

## Additional Information

**How to cite this article**: Zhang, G. *et al*. Bisphosphonates enhance antitumor effect of EGFR-TKIs in patients with advanced EGFR mutant NSCLC and bone metastases. *Sci. Rep.* **7**, 42979; doi: 10.1038/srep42979 (2017).

**Publisher's note:** Springer Nature remains neutral with regard to jurisdictional claims in published maps and institutional affiliations.

## Supplementary Material

Supplemental Table S1

Supplemental Table S2

## Figures and Tables

**Table 1:** Clinical and molecular characteristics of included patients.

	No. of patients (n = 552)	%	TKI + Bps (n = 111)	%	TKI (n = 94)	%	*P* value
Age at diagnosis							0.890
<65 years	357	64.7%	71	64.0%	61	64.9%	
≥65 years	195	35.3%	40	36.0%	33	35.1%	
Gender							0.686
Male	288	52.2%	36	32.4%	33	35.1%	
Female	264	47.8%	75	67.6%	61	64.9%	
Smoking history							0.378
Never-smoker	396	71.7%	98	88.3%	79	84.0%	
Former/current smoker	156	28.3%	13	11.7%	15	16.0%	
ECOG performance status							0.485
0–1	504	91.3%	101	91.0%	88	93.6%	
≥2	48	8.7%	10	9.0%	6	6.4%	
Pathological classification							0.549
Adenocarcinoma	487	88.2%	103	92.8%	90	95.7%	
Non-adenocarcinoma	65	11.8%	8	7.2%	4	4.3%	
SREs							0.147
Yes	143	25.9%	33	29.7%	37	39.4%	
No	409	74.1%	78	70.3%	57	60.6%	
Extra metastases							0.184
Yes	347	62.9%	81	73.0%	76	80.9%	
No	205	37.1%	30	27.0%	18	19.1%	
EGFR mutation							
EGFR mutation	356	64.5%	111	100.0%	94	100.0%	—
EGFR wild type	196	35.5%	0	0.0%	0	0.0%	
BM at time of diagnosis							0.552
Yes	517	93.7%	106	95.5%	88	93.6%	
No	35	6.3%	5	4.5%	6	6.4%	

No., number; BM, bone metastasis; TKI, tyrosine kinase inhibitor; Bps, bisphosphonates; SRE, skeletal related events.

**Table 2:** Univariate and multivariate analyses of clinical parameters in 552 NSCLC patients with BM on overall survival.

Factor	Univariate analysis			Multivariate analysis		
	HR (log rank)	95% CI	*P* value	HR (log rank)	95% CI	*P* value
Gender (Female/Male)	0.966	0.259–1.249	0.574			
Age (<65/ ≥ 65)	0.798	0.562–1.183	0.263			
Smoking (Never/Smoking)	0.921	0.697–1.301	0.255			
Histology (Adeno/Non-adeno)	0.898	0.673–1.242	0.449			
PS (0–1/ > 1)	0.352	0.179–0.811	0.018	0.573	0.192–0.961	0.038
SRE (No/Yes)	0.764	0.382–0.921	0.044	0.863	0.559–1.379	0.353
Number of BM (1/ > 1)	0.889	0.193–5.951	0.983			
Metastasis (B/B + E)	0.545	0.166–1.973	0.289			
EGFR status (Mutation/wild type)	0.632	0.497–0.805	0.003	0.710	0.597–0.913	0.021

HR: hazard ratio; CI: confidence interval; Adeno: adenocarcinoma; BM: bone metastasis; No.: number B: bone; B + E: bone and extra metastasis; PS: performance score; SRE: skeletal related event.

## References

[b1] Liu Y. . Genomic heterogeneity of multiple synchronous lung cancer. Nat Commun. 7, 13200 (2016). 27767028 10.1038/ncomms13200 PMC5078731

[b2] Miller K. D. . Cancer treatment and survivorship statistics, 2016. CA Cancer J Clin. (2016). 10.3322/caac.21349 27253694

[b3] SEER Stat Fact Sheets: Lung and Bronchus Cancer. In. National Cancer Institute 2015.

[b4] Sugiura H., Yamada K., Sugiura T., Hida T. & Mitsudomi T. Predictors of survival in patients with bone metastasis of lung cancer. Clin Orthop Relat Res. 466, 729–736 (2008). 18196360 10.1007/s11999-007-0051-0 PMC2505203

[b5] Vicent S., Perurena N., Govindan R. & Lecanda F. Bone metastases in lung cancer. Potential novel approaches to therapy. Am J Respir Crit Care Med. 192, 799–809 (2015). 26131844 10.1164/rccm.201503-0440SO

[b6] Oliveira M. B., Mello F. C. & Paschoal M. E. The relationship between lung cancer histology and the clinicopathological characteristics of bone metastases. Lung Cancer. 96, 19–24 (2016). 27133744 10.1016/j.lungcan.2016.03.014

[b7] Hendriks L. E., Hermans B. C., van den Beuken-van Everdingen M. H., Hochstenbag M. M. & Dingemans A. M. Effect of Bisphosphonates, Denosumab, and Radioisotopes on Bone Pain and Quality of Life in Patients with Non-Small Cell Lung Cancer and Bone Metastases: A Systematic Review. J Thorac Oncol. 11, 155–173 (2016). 26718881 10.1016/j.jtho.2015.10.001

[b8] Roato I. Bone metastases: When and how lung cancer interacts with bone. World J Clin Oncol. 5, 149–155 (2014). 24829862 10.5306/wjco.v5.i2.149 PMC4014787

[b9] Kuchuk M. . The incidence and clinical impact of bone metastases in non-small cell lung cancer. Lung Cancer. 89, 197–202 (2015). 26003503 10.1016/j.lungcan.2015.04.007

[b10] Daniele S. . Natural History of Non-Small-Cell Lung Cancer with Bone Metastases. Sci Rep. 5, 18670 (2015). 26690845 10.1038/srep18670 PMC4687045

[b11] Tsuya A., Kurata T., Tamura K. & Fukuoka M. Skeletal metastases in non-small cell lung cancer: a retrospective study. Lung Cancer. 57, 229–232 (2007). 17451841 10.1016/j.lungcan.2007.03.013

[b12] Henry D. H. . Randomized, double-blind study of denosumab versus zoledronic acid in the treatment of bone metastases in patients with advanced cancer (excluding breast and prostate cancer) or multiple myeloma. J Clin Oncol. 29, 1125–1132 (2011). 21343556 10.1200/JCO.2010.31.3304

[b13] Scagliotti G. V. . Overall survival improvement in patients with lung cancer and bone metastases treated with denosumab versus zoledronic acid: subgroup analysis from a randomized phase 3 study. J Thorac Oncol. 7, 1823–1829 (2012). 23154554 10.1097/JTO.0b013e31826aec2b

[b14] Li Y. Y. . Zoledronic acid is unable to induce apoptosis, but slows tumor growth and prolongs survival for non-small-cell lung cancers. Lung Cancer. 59, 180–191 (2008). 17900752 10.1016/j.lungcan.2007.08.026

[b15] Lu S. . Synergistic inhibitory activity of zoledronate and paclitaxel on bone metastasis in nude mice. Oncol Rep. 20, 581–587 (2008). 18695909

[b16] Li Y. Y. . Zoledronic acid induces cell-cycle prolongation in murine lung cancer cells by perturbing cyclin and Ras expression. Anticancer Drugs. 22, 89–98 (2011). 20926944 10.1097/CAD.0b013e3283400a05

[b17] Ory B. . Zoledronic acid suppresses lung metastases and prolongs overall survival of osteosarcoma-bearing mice. Cancer. 104, 2522–2529 (2005). 16270320 10.1002/cncr.21530

[b18] Chang J. W. . Bisphosphonate zoledronic acid enhances the inhibitory effects of gefitinib on EGFR-mutated non-small cell lung carcinoma cells. Cancer Lett. 278, 17–26 (2009). 19233551 10.1016/j.canlet.2008.12.019

[b19] Huang C. Y. . Bisphosphonates enhance EGFR-TKIs efficacy in advanced NSCLC patients with EGFR activating mutation: A retrospective study. Oncotarget. (2015). 10.18632/oncotarget.5515 PMC5341815 26624882

[b20] Melisi D. . Zoledronic acid cooperates with a cyclooxygenase-2 inhibitor and gefitinib in inhibiting breast and prostate cancer. Endocr Relat Cancer. 12, 1051–1058 (2005). 16322342 10.1677/erc.1.01061

[b21] Zarogoulidis K. . The impact of zoledronic acid therapy in survival of lung cancer patients with bone metastasis. Int J Cancer. 125, 1705–1709 (2009). 19521984 10.1002/ijc.24470

[b22] Hirsh V. . Zoledronic acid and survival in patients with metastatic bone disease from lung cancer and elevated markers of osteoclast activity. J Thorac Oncol. 3, 228–236 (2008). 18317064 10.1097/JTO.0b013e3181651c0e

[b23] Pandya K. J. . Multicenter, randomized, phase 2 study of zoledronic acid in combination with docetaxel and carboplatin in patients with unresectable stage IIIB or stage IV non-small cell lung cancer. Lung Cancer. 67, 330–338 (2010). 19493585 10.1016/j.lungcan.2009.04.020

[b24] Murakami H. . Phase II study of zoledronic acid combined with docetaxel for non-small-cell lung cancer: West Japan Oncology Group. Cancer Sci. 105, 989–995 (2014). 24837137 10.1111/cas.12448 PMC4317856

[b25] Wood S. L., Pernemalm M., Crosbie P. A. & Whetton A. D. The role of the tumor-microenvironment in lung cancer-metastasis and its relationship to potential therapeutic targets. Cancer Treat Rev. 40, 558–566 (2014). 24176790 10.1016/j.ctrv.2013.10.001

[b26] Roodman G. D. Mechanisms of bone metastasis. N Engl J Med. 350, 1655–1664 (2004). 15084698 10.1056/NEJMra030831

[b27] Jones D. H. . Regulation of cancer cell migration and bone metastasis by RANKL. Nature. 440, 692–696 (2006). 16572175 10.1038/nature04524

[b28] Peters S. & Meylan E. Targeting receptor activator of nuclear factor-kappa B as a new therapy for bone metastasis in non-small cell lung cancer. Curr Opin Oncol. 25, 137–144 (2013). 23283210 10.1097/CCO.0b013e32835d720b

[b29] Sawant A. & Ponnazhagan S. Myeloid-derived suppressor cells as osteoclast progenitors: a novel target for controlling osteolytic bone metastasis. Cancer Res. 73, 4606–4610 (2013). 23887974 10.1158/0008-5472.CAN-13-0305 PMC3732563

[b30] Travis W. D. . International association for the study of lung cancer/american thoracic society/european respiratory society international multidisciplinary classification of lung adenocarcinoma. J Thorac Oncol. 6, 244–285 (2011). 21252716 10.1097/JTO.0b013e318206a221 PMC4513953

[b31] Zhao M. . The Bim deletion polymorphism clinical profile and its relation with tyrosine kinase inhibitor resistance in Chinese patients with non-small cell lung cancer. Cancer. 120, 2299–2307 (2014). 24737648 10.1002/cncr.28725

[b32] Wu C. . High Discrepancy of Driver Mutations in Patients with NSCLC and Synchronous Multiple Lung Ground-Glass Nodules. J Thorac Oncol. 10, 778–783 (2015). 25629635 10.1097/JTO.0000000000000487

[b33] Li W. . T790M mutation is associated with better efficacy of treatment beyond progression with EGFR-TKI in advanced NSCLC patients. Lung Cancer. 84, 295–300 (2014). 24685306 10.1016/j.lungcan.2014.03.011

[b34] Li J. . miR-200c overexpression is associated with better efficacy of EGFR-TKIs in non-small cell lung cancer patients with EGFR wild-type. Oncotarget. 5, 7902–7916 (2014). 25277203 10.18632/oncotarget.2302 PMC4202169

[b35] Jiang T. . EGFR TKIs plus WBRT demonstrated no survival benefit than TKIs alone in NSCLC patients with EGFR mutation and brain metastases. J Thorac Oncol. (2016). 10.1016/j.jtho.2016.05.013 27237825

[b36] De Marinis F. . Bisphosphonate use in patients with lung cancer and bone metastases: recommendations of a European expert panel. J Thorac Oncol. 4, 1280–1288 (2009). 19701109 10.1097/JTO.0b013e3181b68e5a

[b37] Sun Y. . [Expert consensus on the diagnosis and treatment of bone metastasis in lung cancer (2014 version)]. Zhongguo Fei Ai Za Zhi. 17, 57–72 (2014). 24581154 10.3779/j.issn.1009-3419.2014.02.01 PMC6000054

